# Peripheral vascular access for catheter ablation of supraventricular tachycardia using remote magnetic navigation

**DOI:** 10.1016/j.hrcr.2021.03.004

**Published:** 2021-03-17

**Authors:** Sabine Ernst, Nelly Samchkuashvili, Suraj Kadiwar, Bruce Barton, Christoph Nienaber, Jan Till

**Affiliations:** ∗ Department of Cardiology, Royal Brompton and Harefield NHS Foundation Trust, National Heart and Lung Institute, Imperial College, London, United Kingdom; † Department of Radiology, National Heart and Lung Institute, Imperial College, Royal Brompton and Harefield Hospital, London, United Kingdom; ‡ Department of Paediatric Cardiology, Royal Brompton and Harefield NHS Foundation Trust, National Heart and Lung Institute, Imperial College, London, United Kingdom

**Keywords:** Catheter ablation, Congenital heart disease, Radiation exposure, Remote magnetic navigation, Supraventricular tachycardia, Vascular access

## Introduction

Catheter ablation has traditionally been performed via femoral vascular access.^1^ Diagnostic and therapeutic catheters are typically preformed or steerable such that they can be positioned in key sites—for example, at the His recording region (His) or coronary sinus. Manipulation of such catheters can be demanding from alternative sites. Remote magnetic navigation allows the operator to navigate the tip of a mapping and ablation catheter in all degrees of freedom, such that the access route is no longer relevant during the procedure.^2^^,^^3^ In the last 2 decades, coronary interventions are increasingly carried out via a brachial or radial approach, with most of these procedures now performed without puncturing the femoral artery. Shorter recovery times, improved cost-effectiveness, and fewer local complications are reported consequences.^4^^,^^5^

We report on catheter ablation procedures in 2 patients carried out via peripheral vascular access assisted by remote magnetic navigation and 3D mapping for right- and left-sided arrhythmia substrates.

## Case report

**Figure 1:**
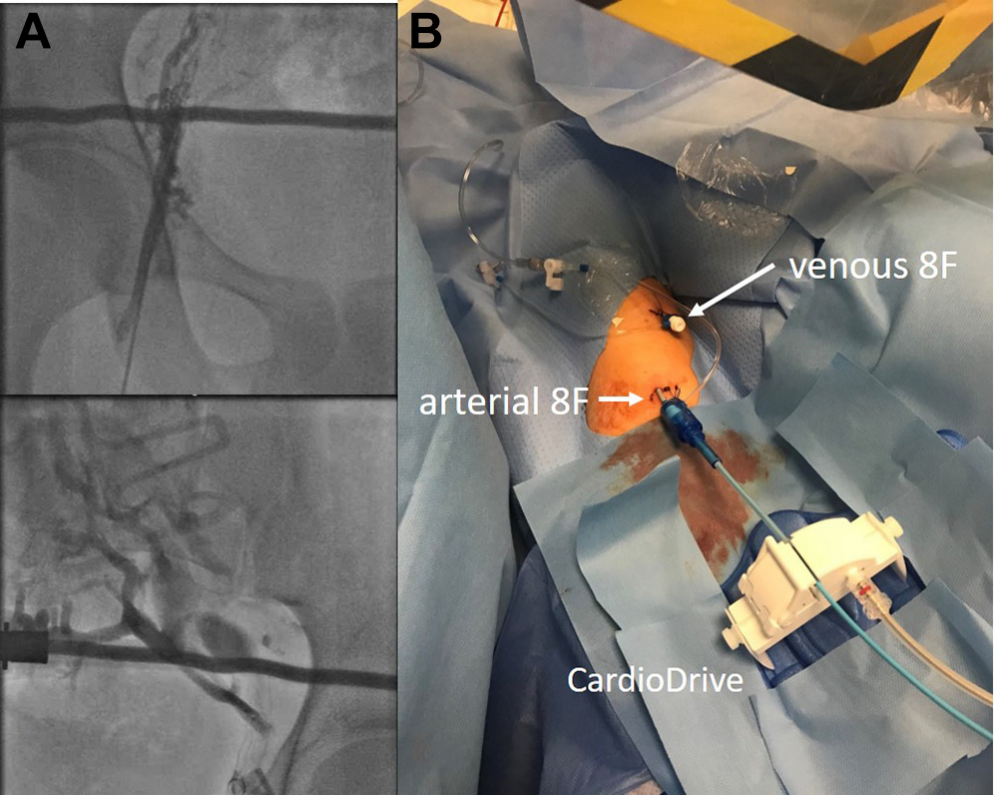
**A:** Depiction of blocked femoral vessels of patient 2 by direct contrast injection. **B:** Peripheral vascular accesses of the right arm in patient 2 with both venous and arterial 8F sheaths inserted.

Two male patients (aged 42 and 17 years) each presented with problematic supraventricular arrhythmia and failed conventional catheter ablation procedures owing to congenital absence of an inferior caval vein (patient 1) or inaccessible bilateral femoral venous access (patient 2) (Figure 1A). Patient 1 with tricuspid atresia and pulmonary stenosis had undergone a right Blalock-Tausig shunt in 1978 and pulmonary artery Fontan repair in 1988. Patient 2 was diagnosed with Wolff-Parkinson-White syndrome and frequent supraventricular tachycardia from birth; suffered from necrotizing colitis in his first week of life, requiring an ileostomy; and required prolonged pediatric intensive care.

### Peripheral vascular access

Using an ultrasound-guided vascular access technique, both left and right brachial veins were accessed and conventional 8F short sheath was positioned (8F, 11 cm, Avanti+, Cordis, Florida).^6^ For arterial access in patient 2, a 4F sheath (Avanti+; Cordis, Hialeah, FL) was positioned in the right brachial artery in the same fashion and was eventually upsized to 8F (Figure 1B).

### Invasive electrophysiological study

**Figure 2:**
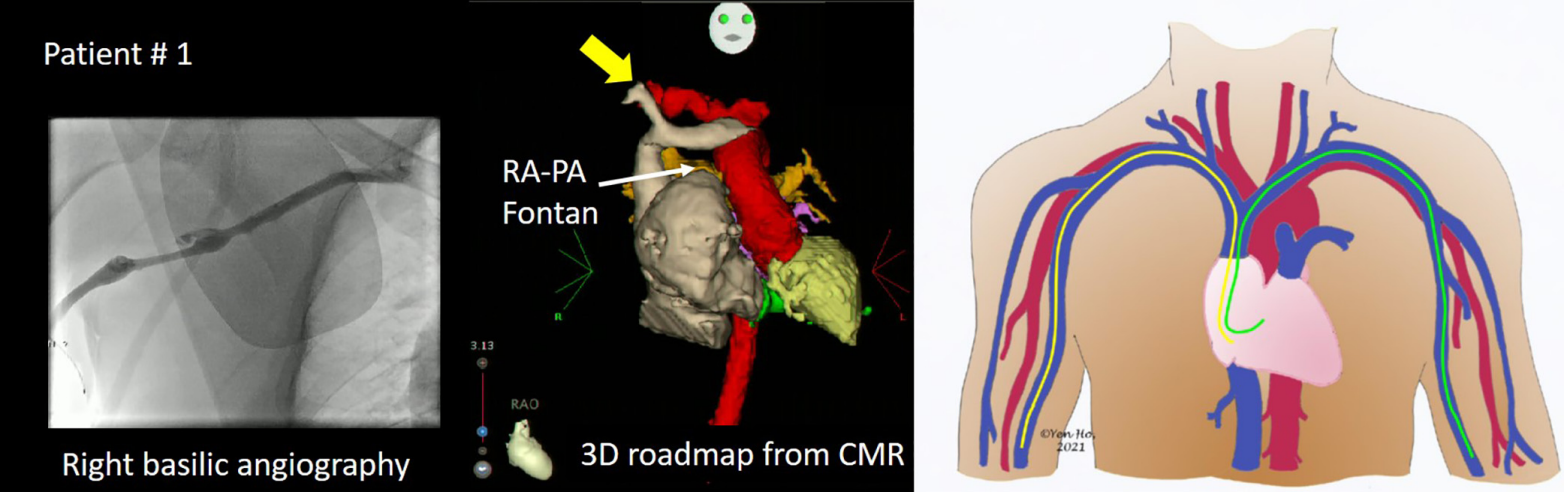
Arrhythmia substrates for both patients. **Left:** The contrast injection in the right basilic vein. **Middle:** The 3D reconstruction from cardiac magnetic resonance (CMR). **Right:** A schematic of the paths of the reference (from the left arm, *green*) and the magnetic ablation catheter (from the right arm, *yellow*) which was identical for both patients (courtesy of Prof. Ho, Royal Brompton Hospital, London, UK).

The diagnostic catheter was positioned under fluoroscopy guidance from the left brachial access in both cases; a decapolar steerable catheter (6F, Dynamic tip; Boston Scientific, Marlborough, MA) in the proximal coronary sinus in patient 1 and a nonsteerable octapolar catheter (6F, Inquiry, IBI-80043; St. Jude Medical, Zaventem, Belgium) in patient 2 (Figure 2). In both cases, the electroanatomical mapping system CARTO was used with an irrigated-tip magnetic navigation catheter (NaviStar RMT irrigated; Biosense Webster, Brussels, Belgium) in combination with a mechanical Cardiodrive unit (Stereotaxis Inc, St. Louis, MO). The magnetic catheter was advanced from the right brachial vessels (Figure 2). Three-dimensional image integration from cardiac magnetic resonance imaging was used as a 3D roadmap in patient 1 (Figure 2).

Access to the right atrium and subsequent mapping of the arrhythmia substrate was successful in both patients and depicted a reentrant tachycardia around an atretic tricuspid annulus in patient 1. A linear lesion was deployed from the absent tricuspid annulus to the inferior caval vein, which terminated the atrial tachycardia and rendered the patient noninducible.

In patient 2, a left lateral bidirectionally conducting accessory pathway was located in left lateral position. The magnetic catheter was subsequently advanced via the peripheral arterial access and retrogradely advanced into the left ventricle and also the left atrium (Figure 1, right panel). Catheter ablation was carried out in using 45 watts (flow rate of 30 mL/min) and blocked the accessory pathway within 4 seconds.

No further arrhythmia was inducible in either of the patients and the procedures were terminated after a waiting time of 45 minutes, respectively. All sheaths were removed, and manual pressure was applied for hemostasis.

**Figure 3:**
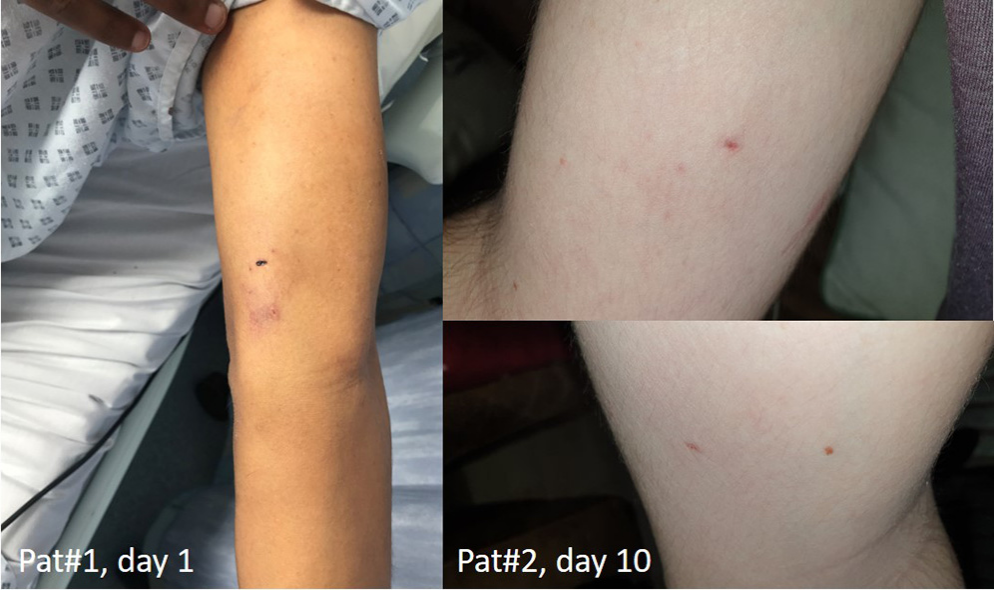
Photographs of the peripheral vascular access sites of patient 1 (left, venous only, day 1) and patient 2 (right, both venous and arterial, day 10) postprocedurally.

Total procedure time amounted to 178 and 120 minutes with 2.54 and 0.54 minutes of fluoroscopy, respectively. Both patients were able to immediately mobilize and were monitored overnight on telemetry. After exclusion of any relevant pericardial effusion, they were discharged on the following day. The vascular access sites were reviewed and followed up by video clinic (Figure 3).

## Discussion

To our knowledge, this is the first report of successful ablation procedures via peripheral vascular accesses in patients with blocked femoral venous access. While there have been reports of ablation procedures carried out from a superior approach via subclavian or jugular access, these procedures always pose the risk of local access complications such as iatrogenic pneumothorax or hemothorax.^7^, ^8^, ^9^, ^10^, ^11^

Currently vascular access for diagnostic procedures such as right heart catheters or for long-term peripheral central access lines are standard techniques and have excellent success rates and very low long-term complication risk, even when lines remain within the patients for many weeks and months.^12^^,^^13^ Careful ultrasound puncture technique is key to allow for precise and uncomplicated access.^6^ Increasingly, patients with congenital heart disease / neonatal issues are living longer. The advantages of peripheral access sites over the more central ones are clear, but with conventional navigation techniques manipulation of the ablation catheter would be challenging. These difficulties can be overcome by the use of remote magnetic navigation, as the ablation catheter is steered by the outer magnetic field interacting with magnets on the catheter tip while the shaft of the catheter is very soft and floppy, allowing for multiple curves without loss of catheter tip control,^14^ thus allowing ease of access in patients with blocked femoral access.

### Future implications

Using peripheral vascular accesses has the advantage that the patient is immediately mobilized and restriction for physical activities are not necessary, thereby substantially shortening the recovery time. In addition, the reduction of the number of catheters needed (single diagnostic plus magnetic ablation catheter) has potential cost-saving implications.

Our technique offers an alternative option to enable electrophysiologic procedures to be performed from exclusively peripheral access sites. This could potentially transform electrophysiological procedures in a similar way as interventional procedures, which moved from femoral to radial access in recent years.

## Conclusion

Two patients with blocked femoral vascular access underwent successful catheter ablation using remote magnetic navigation via vascular access from both arms. This technique excludes the risk for pneumothorax, allows immediate mobilization, and was successfully used for both right- and left-sided arrhythmias.

**Key Teaching Points**

• Catheter ablation can be safely and effectively be carried out via vascular access from the arms.
• This novel peripheral access route is an alternative in patients with blocked or absent inferior access.
• This technique requires remote magnetic navigation to allow versatile navigation of the ablation catheter.
• To avoid injury at the access sites vascular access should be performed using ultrasound guidance.
